# A Survey of Older Adults’ Perspectives of In-Person and Virtual Parkinson’s-Specific Exercise Classes

**DOI:** 10.1093/geroni/igaa057.3416

**Published:** 2020-12-16

**Authors:** Holly Bennett, Jennifer Vincenzo, Chris Oholendt

**Affiliations:** University of Arkansas for Medical Sciences, Little Rock, Arkansas, United States

## Abstract

Due to COVD-19, many health/wellness programs transitioned from in-person to virtual. This mixed methods study aims to explore older adults with Parkinson’s disease (PD) perceptions of in-person versus virtual Parkinson’s-specific exercise classes. Attitudes, perceptions, and experiences were determined through focus groups (n=9; Male=4; aged 75 years) among older adults with PD and an online survey (n=23; Male=14; aged 74 years). Eighteen respondents attended both in-person and virtually (n=18; Male=9). Four respondents only attended in-person, citing reasons such as difficulty with computer access to virtual classes, limited internet, easier accessibility to in-person classes, and physical injury preventing attendance to any classes. Respondents who participated in both delivery methods preferred virtual classes. Time, convenience, comfort at home, and not having to navigate transportation barriers supported participants’ preference for virtual classes. The majority of respondents indicated their fatigue and mental health were either unchanged or improved. Eighty-nine percent reported improved mobility since attending either class; specifically, in balance (n=8), flexibility (n=7), and coordination (n=3). Older adults with PD who attended both classes had minimal difficulty with computer usage and accessing the virtual program with only one participant reporting difficulty transitioning from in-person to virtual classes. Seventy percent stated they would enjoy a combination of on-site and virtual programming. Eighty-nine percent and seventy-seven perfect felt the virtual-based program was safe and beneficial, respectively. Participants who transitioned from an in-person to a virtual exercise program for people with Parkinson’s disease felt the program was safe, effective, and improved or prevented declines in their mobility.

